# Gaussian Process Emulation for Modeling Dengue Outbreak Dynamics

**DOI:** 10.1101/2024.11.28.24318136

**Published:** 2024-11-29

**Authors:** Anna M. Langmüller, Kiran A. Chandrasekher, Benjamin C. Haller, Samuel E. Champer, Courtney C. Murdock, Philipp W. Messer

**Affiliations:** 1 Department of Computational Biology, Cornell University, Ithaca, NY 14853, USA; 2 Department of Mathematics, University of Vienna, Vienna 1090, Austria; 3 Aarhus Institute of Advanced Studies, Aarhus University, Aarhus C 8000, Denmark; 4 Department of Entomology, Cornell University, Ithaca, NY 14853, USA; 5 Cornell Institute of Host-Microbe Interactions and Disease, Cornell University, Ithaca, NY 14853, USA; 6 Center for the Ecology of Infectious Diseases, University of Georgia, Athens GA, USA

**Keywords:** individual-based modeling, statistical emulation, Gaussian Processes, epidemiological modeling, variance-based sensitivity analysis

## Abstract

Epidemiological models that aim for a high degree of biological realism by simulating every individual in a population are unavoidably complex, with many free parameters, which makes systematic explorations of their dynamics computationally challenging. This study investigates the potential of Gaussian Process emulation to overcome this obstacle. To simulate disease dynamics, we developed an individual-based model of dengue transmission that includes factors such as social structure, seasonality, and variation in human movement. We trained three Gaussian Process surrogate models on three outcomes: outbreak probability, maximum incidence, and epidemic duration. These models enable the rapid prediction of outcomes at any point in the eight-dimensional parameter space of the original model. Our analysis revealed that average infectivity and average human mobility are key drivers of these epidemiological metrics, while the seasonal timing of the first infection can influence the course of the epidemic outbreak. We use a dataset comprising more than 1,000 dengue epidemics observed over 12 years in Colombia to calibrate our Gaussian Process model and evaluate its predictive power. The calibrated Gaussian Process model identifies a subset of municipalities with consistently higher average infectivity estimates, highlighting them as promising areas for targeted public health interventions. Overall, this work underscores the potential of Gaussian Process emulation to enable the use of more complex individual-based models in epidemiology, allowing a higher degree of realism and accuracy that should increase our ability to control important diseases such as dengue.

## Introduction

Simulation models that describe individual organisms or agents have become a well-established research tool across numerous scientific fields^1^. These so-called individual-based models (IBMs) can allow researchers to explore how system-level characteristics emerge from individual behaviors, while also investigating the reciprocal influence of the system on individuals^1^. In the field of epidemiology, IBMs have provided valuable insights into the dynamics of pathogen and disease spread and have facilitated rigorous evaluation of planned intervention strategies, making them an integral part of modern epidemiological research^2–6^. Recent computational advances, combined with the development of comprehensive individual-based simulation frameworks^2,7^, have enabled the creation of epidemiological models with unprecedented realism. These models can capture details ranging from fine-scale human movement^5,8^ to specific larval breeding sites for organisms that spread vector-borne diseases^3^.

While enhanced biological realism has undeniably deepened our understanding of epidemiological processes, it also introduces increased complexity because of the level of detail being simulated, which comes at a substantial computational cost. As IBMs become more realistic, they also become more parameter-rich, making it increasingly difficult to identify the key drivers of disease dynamics. This is partly because parameters often interact in complex, non-linear ways, complicating efforts to quantify the contribution of any single factor to model outcomes. Global sensitivity analysis can help by quantifying the relative contribution of each parameter — as well as their interactions — to IBM outcomes. For example, the Sobol method^9^ is a global sensitivity analysis approach that is able to assess complex, non-linear parameter interactions by partitioning the observed variance in IBM output into relative contributions from single parameters as well as interactions between two or more parameters. This allows researchers to gain a deeper understanding of the key drivers of the disease dynamics observed in a simulation.

Global sensitivity analysis can provide valuable insights into model dynamics, but generating sufficient data for robust sensitivity analysis becomes increasingly computationally demanding with each additional parameter^10^. Even with IBMs optimized for runtime performance, comprehensively surveying high-dimensional parameter spaces can be daunting and time-consuming. This is particularly true when large populations are modeled, since the computational complexity of IBMs typically scales at least linearly with the number of individuals modeled. When more intricate behaviors or interactions are included, the computational burden can even increase quadratically (such as when every pairwise interaction between individuals must be simulated), further intensifying the challenge of parameter space exploration.

Statistical emulation^11^ is a powerful technique for analyzing IBMs with high-dimensional parameter spaces. It can potentially provide an efficient solution to this dimensionality problem (sometimes referred to as the “Curse of Dimensionality”^10^) by greatly reducing the number of simulation runs required to understand the behavior of the IBM across the parameter space of interest. This technique involves the creation of a surrogate model, based on a limited set of IBM simulations, that can rapidly predict the output of the IBM. Emulators can be developed using either statistical, or, more recently, machine learning approaches^12^. A key feature of a well-designed emulator is its ability to provide highly accurate predictions significantly faster than the IBM could — often orders of magnitude faster. In some cases, this can effectively reduce simulation times from days or weeks to mere seconds or minutes, paving the way for comprehensive sensitivity analysis and broader model exploration.

Gaussian Processes (GPs), first introduced in the 1960s within the field of geostatistics^13,14^, are flexible statistical emulators that have been successfully applied across a range of disciplines^15–17^. GPs are non-parametric models that define a distribution over functions based on observed data. A key advantage of GPs over other machine learning techniques, such as conventional support vector machines or neural networks, lies in their Bayesian foundations, which allow GPs to provide confidence intervals alongside their predictions. This uncertainty quantification enables efficient sampling of additional training data from regions with the greatest uncertainty, facilitating active learning^18^ that can quickly produce highly accurate emulation. Furthermore, with the availability of advanced software packages supporting GPU acceleration, the computational efficiency of GPs has improved at an astonishing pace in recent years, making them an increasingly attractive research tool^19^.

In epidemiology, the ability of GPs to efficiently extrapolate between sparse data points is often utilized for estimating disease incidence counts in areas where data is missing or unobserved^20,21^. Beyond interpolation, GPs serve as valuable forecasting tools^17,22^, and are key components of early-warning systems^23^. Furthermore, as emulators of complex, computationally intensive IBMs, GPs facilitate the calibration of these IBMs to empirical data by helping to select parameter values for which the IBM’s outputs closely match observed real-world data^24–26^. However, the potential of GPs for enabling comprehensive sensitivity analysis — particularly to identify key drivers of disease dynamics in an IBM — remains underexplored. Notable examples of using GPs as emulators to better understand complex IBMs include recent studies that applied GP emulation to the OpenMalaria model^26^ (an advanced IBM developed to simulate malaria transmission and control) to explore key drivers of the spread of drug-resistant *Plasmodium falciparum*^27^, and to assess the effectiveness of various intervention strategies^28,29^. However, these previous studies focused exclusively on malaria.

In this study, we introduce the use of GP-based sensitivity analysis to dengue transmission modeling, offering a refined perspective on the factors that drive epidemic patterns. We employed GP surrogate models to efficiently predict three key metrics from a dengue-inspired individual-based disease transmission model: outbreak probability, maximum incidence, and duration. Dengue poses a growing global health threat^30^, with cases rapidly increasing due to urbanization^31^ and climate change that has expanded the habitat of *Aedes* mosquitoes, the primary vectors of the dengue virus^32,33^. Our IBM allows us to simulate disease transmission while accounting for social structure, human movement, and variation in infection probability. We demonstrate that our GPs explore the parameter space with impressive speed, enabling a more comprehensive sensitivity analysis than previous studies have undertaken. We conduct a global sensitivity analysis and find that average infectivity and average human mobility are primary drivers of outbreak dynamics, while the interactions between seasonality strength and initial infection timing can critically influence the course of epidemic outbreaks. To determine whether insights from our sensitivity analysis could inform our understanding of real-world epidemics, we investigate weekly dengue incidence data at the municipality level across Colombia over more than a decade^34,35^, identifying municipalities that could serve as potential candidates for targeted interventions or in-depth studies.

## Results

### Individual-based model

We implemented an IBM in C++ that simulates and tracks transmission dynamics of dengue to study how disease dynamics are influenced by infection probability, human movement, and social structure. The model is designed not to replicate a specific empirical system, but to illustrate the relative importance of these parameters and their interactions in influencing the course of epidemic outbreaks. A detailed description of the IBM can be found in the Materials & Methods section. All model parameters are summarized in Table 1. Briefly, each simulation begins by generating 10,000 locations that are each home to a group of susceptible individuals. The number of individuals per location is sampled from a negative binomial distribution fitted to the demography of Iquitos, Peru — a well-studied dengue transmission hotspot^36,37^. These locations are then randomly organized into non-overlapping family clusters, with the “family cluster size” parameter controlling the number of locations per cluster. Social structure, controlled by the “social structure” parameter, influences the likelihood that individuals interact within their family cluster. Human movement — the number of visits to locations per day — is sampled from a negative binomial distribution, defined by the “average mobility” and “mobility skewness” parameters. The social structure of the model is depicted in Supplemental Figure S1.

The disease is introduced by infecting a single randomly chosen individual. Infected individuals remain contagious for a number of days specified by the “infectious period” parameter, after which they recover and gain lasting immunity (and thus cannot become reinfected). When a susceptible individual visits a location that was visited by infectious individuals the day before, the likelihood of infection from each previous infectious visitor is determined by the infection probability, which accounts for seasonal fluctuations in infection risk due to changes in mosquito abundance. We chose not to model individual mosquitoes explicitly. This choice was driven by the very limited dispersal ability of *Aedes aegypti* ^38^, the primary vector for dengue transmission in the Americas, which predominantly bites during daylight hours^39^. Consequently, human movement patterns tend to be more influential than mosquito movement in shaping dengue dynamics^36,40,41^.

The infection probability is defined by a cosine function with three parameters: (i) the “average infectivity” parameter (α_0_), representing the average infection probability over the course of a year (365 days); (ii) the “seasonality strength” parameter (α_*season*_), controlling the magnitude of seasonal variation in infection probability; and (iii) the “first case timing” parameter (*t_first_*), defining the horizontal shift of the cosine function and thus the timing of the first case relative to the peak infection probability due to seasonality. Together, these parameters define the infection probability at any given day *t* in the year:

$$p_{\text{infection}}(t)={\alpha_{0}}^{*}\left(1+\alpha_{\text{season}}*\cos\left(2\pi *\left(t/365-t_{\text{first}}\right)\right)\right)$$

The IBM progresses by daily timesteps and continues until there are no infectious individuals left. The output consists of daily counts of individuals in each infection state (susceptible, exposed, infectious, and recovered). For each combination of parameters, we use 100 replicate simulation runs to calculate three metrics of the simulated epidemics: (i) outbreak probability, defined as the proportion of simulation runs in which more than 0.1% of the population becomes infected; (ii) maximum disease incidence (*i*_*max*_), defined as the highest proportion of infectious individuals seen in any timestep; and (iii) outbreak duration, defined as the timespan in days from the first infectious case to the recovery of the last infectious individual. To calculate *i*_*max*_ and outbreak duration, we average across 100 simulation runs where an outbreak occurred (doing additional runs as needed to obtain 100 such runs for each parameter combination), thereby minimizing confounding effects from stochastic losses of the disease.

We systematically varied the eight parameters outlined above to explore how the simulated epidemics change across the parameter space. Across the full range of parameters (Table 1), the three metrics exhibit significant variability: the average outbreak probability is 0.79, ranging from 0 to 1; the average *i*_*max*_ is 0.67, ranging from 0.0003 to 0.99; and the average duration is 63.88 days, ranging from 19.65 to 424.15 days.

### Gaussian Process training & performance

**Figure 1.**
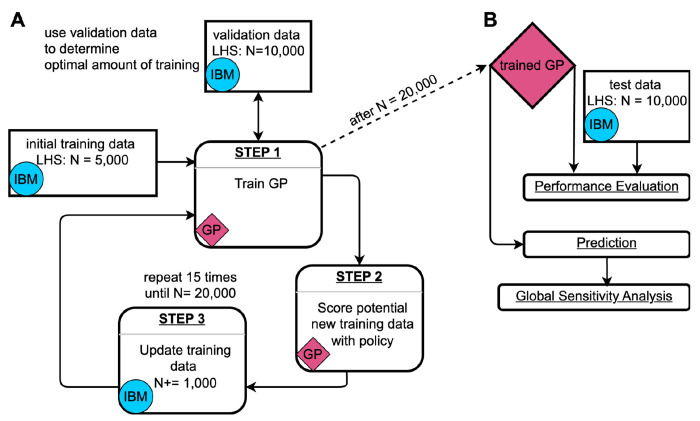
(A) Gaussian Process (GP) training loop^18^. The GP training begins with an initial training dataset consisting of a Latin hypercube sample (LHS) of 5,000 data points generated from the input domain (Table 1) using the individual-based simulation model (IBM). During training, the GP is continuously evaluated against a validation dataset of 10,000 data points to prevent overfitting. After each training cycle, 10^7^ potential new data points are scored based on a policy that considers their predicted value and 95% confidence interval. In each iteration of the training loop, 1,000 additional data points are sampled from those 10^7^ candidate points, where the probability of being sampled is proportional to their respective scores. The newly selected data points are then simulated using the IBM, added to the training dataset, and the next training iteration begins. (B) Usage of the trained GP. After training, the GP is tested using an independent dataset of 10,000 LHS data points to evaluate its performance. The trained GP can then be used for rapid predictions, enabling large-scale global sensitivity analyses.

Even for relatively simple IBMs, generating the necessary data for a comprehensive sensitivity analysis can become computationally prohibitive, especially when the parameter space is high-dimensional and parameters interact with one another in complex ways. To address this, we implemented GP surrogate models as statistical emulation tools^11,12^, and trained them on input–output data pairs from our IBM (Materials & Methods section). We trained three independent GPs to predict outbreak probability, *i*_*max*_, and outbreak duration. The Bayesian nature of GPs allows for uncertainty quantification of single predictions^18^, which we utilized in an active learning loop to adaptively select additional training data points (Figure 1A).

Thanks to the optimization techniques and GPU acceleration implemented in GPyTorch^19^, GP training remained computationally manageable. On our local machine (i5-12600K CPU, GeForce RTX 4090 GPU), one training round with 30,000 iterations took approximately 15 minutes to 2 hours, depending on the size of the training data (5,000 – 20,000 points, Figure 1A: Step 1). The majority of time in the overall process was spent generating additional training data with the IBM during the active learning rounds (Figure 1A: Step 3). This was more time-intensive (~10 hours on our local machine) for the GPs modeling *i*_*max*_ and outbreak duration than for the GP modeling outbreak probability, because the training data for the latter also included many simulation runs where no outbreaks occurred (which are typically very fast).

One of the primary goals of statistical emulation is to reduce computational time^12^. GPs are particularly efficient for this purpose because they provide closed-form solutions for predictions, and computationally expensive matrix inversions are performed during training, not during prediction^18,42^. Thus, once trained, GPs can predict new data points rapidly. In our case, we observed a significant speed-up: with our local machine we were able to predict the 10,000 data points needed for Figure 3C in only 0.1 seconds. This is equivalent to performing 10^6^ IBM simulation runs, which, depending on available computational resources, would typically take several hours in a computing cluster environment. Additionally, the runtime for GP predictions is deterministic and constant due to the closed-form solution, whereas the runtime of IBM simulations varies depending on the duration of the disease outbreak.

Model performance was evaluated using root mean square error (RMSE) — both for continuous checks against a validation dataset during training to avoid overfitting (Figure 1A), and for assessing the accuracy of the GPs after training was completed (Figure 1B). During training, we observed that the first few adaptive training rounds tended to lead to the most significant improvements in model performance, whereas additional rounds later in the process yielded diminishing returns (Figure S1). The final GPs achieved RMSE values of 0.057 for outbreak probability, 0.042 for *i*_*max*_, and 0.068 for duration (Figure S2).

**Figure 2.**
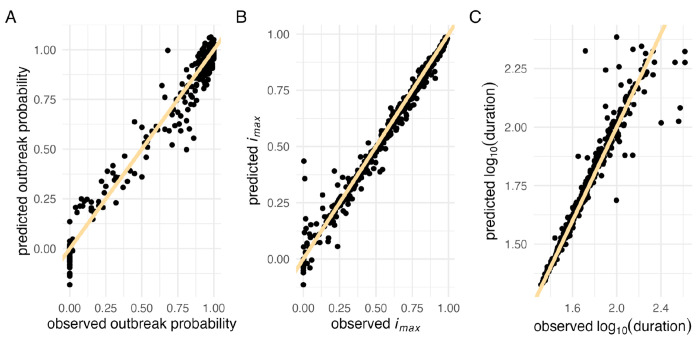
Gaussian Process performance evaluation. Comparison of observed versus predicted values for 500 randomly sampled test data points. The yellow line represents the identity line (*x* = *y*) for (A) outbreak probability (B) maximum incidence (*i_max_*), and (C ) log_10_-transformed duration.

We observed greater variance in the model’s predictive accuracy for weaker epidemics (i.e., lower *i*_*max*_ values, Figure 2B) and longer epidemics (i.e., larger duration, Figure 2C). In such cases, stochasticity plays a larger role, making predictions more challenging. For the *i*_*max*_ GP, there were instances where the intensity of the epidemic outbreak was severely overpredicted (Figure 2B). This might have resulted from neighboring data points with very different properties. Although our kernel choice allows for rather abrupt changes in function values, the interpolation might not fully capture the true dynamics of the underlying model if it does not happen exactly at the midpoint between the two points.

### Sensitivity analysis & specific model outcomes

The computational efficiency of the GPs allowed us to perform a comprehensive variance-based sensitivity analysis of our model. For this, we used the Sobol method^9^, a variance-based approach that quantifies the contribution of each variable — both independently and in interaction with others — to the overall variance of the model output. We estimated first-order effects (the influence of single parameters independent of others), second-order effects (the contribution of pairwise interactions between parameters), and total-order effects (which include first-order effects as well as all interactions of any order).

**Figure 3.**
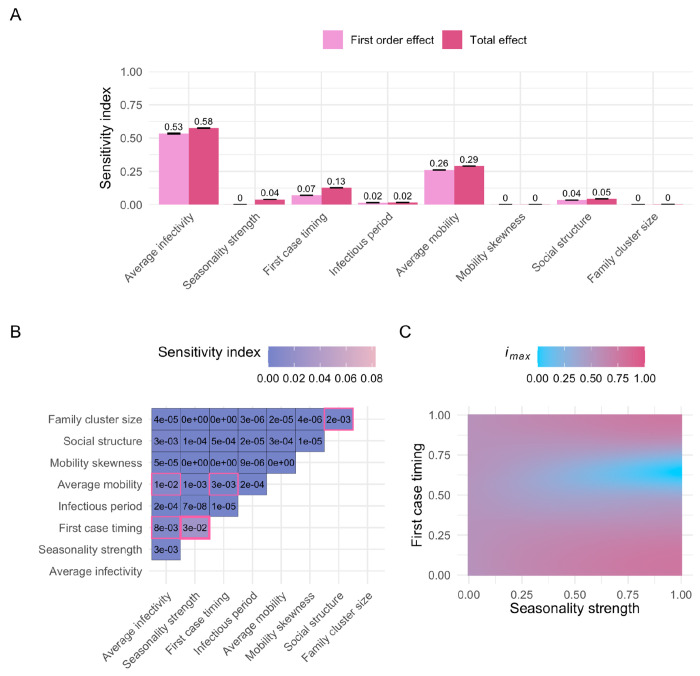
Sobol sensitivity analysis, maximum incidence (*i_max_*). (A) First-order and total effects across the entire input domain (Table 1). The first-order effect describes the impact of a single parameter on the model output (*i_max_*), while the total effect of a parameter accounts for both its first-order effect and all interactions with other parameters. Error bars represent the 95% confidence intervals of the sensitivity index estimates. A total of 9,437,184 points were evaluated for the sensitivity analysis. (B) Second-order effects across the entire input domain (Table 1). A second-order effect captures the pairwise interaction between two parameters. Sobol indices with a 95% confidence interval that does not overlap zero are highlighted with a pink border. The largest second-order effect is emphasized with a bold pink border. (C) *i_max_* predictions with varying “seasonality strength” and “first case timing” parameters (i.e., the two parameters with the largest second-order effect, see panel B). Other parameters were fixed at default values (Table 1). Corresponding sobol sensitivity analysis plots for outbreak probability and outbreak duration can be found in the supplementary figure section (Figures S3 and S4).

This analysis revealed that average infectivity and average mobility are the primary drivers in our model, shaping all three epidemiological metrics: outbreak probability, *i*_*max*_, and outbreak duration. Since there is no correlation between the number of visits sampled for a given individual over time, our model does not include systematic super-spreading behavior. As a result, we expected the sensitivity index for mobility skewness to be low across all three metrics, in contrast to a stronger impact of average mobility. Our findings confirm this expectation, with mobility skewness showing no influence on the epidemiological metrics (*i_max_*: Figure 3A, outbreak probability: Figure S3A, outbreak duration: Figure S4A).

The first-order effect estimates for average infectivity are nearly identical across all three metrics: outbreak probability, *i*_*max*_, and duration (0.52, 0.53, and 0.53, respectively; Figures 3, S3–S4). However, the total effect of average infectivity is notably higher for outbreak probability (0.69; Figure S3) compared to *i*_*max*_ and duration (both 0.58; Figure 3, S4). The higher total-order effect for outbreak probability is primarily driven by the interaction between average infectivity and average mobility (Figure S3B–C). This makes intuitive sense, as highly infectious diseases can still trigger outbreaks even when individual movement is limited. By contrast, the spread of less infectious diseases relies more heavily on sufficient individual mobility to compensate for a lower per-contact infection probability. Accordingly, the first-order effects of average mobility are smaller for outbreak probability compared to *i*_*max*_ and duration (0.12 vs. 0.26 and 0.27). However, the total-order effects of average mobility are similar across all three metrics (0.27, 0.29, and 0.3, respectively, Figures 3, S3–S4).

The reduced importance of the interaction between average infectivity and average mobility for *i*_*max*_ and duration is due to the fact that these metrics are calculated only across simulations in which an actual outbreak occured. For these two metrics, the largest second-order effect is the interaction between the seasonality strength and the timing of the first infectious case (Figure 3B, Figure S4B). In our model, the infection probability fluctuates seasonally, following a cosine pattern, and thus the initial infection timing relative to the seasonal cycle is crucial. Introducing the disease during a low-risk season (i.e., when the value of the first case timing parameter falls between 0.25 and 0.75) can lead to prolonged epidemics with lower *i*_*max*_ (Figure 3C, Figure S4C). Since the infection probability depends on the interaction between the average infectivity, the seasonality strength, and the first case timing, it is encouraging that sensitivity analyses of the GP surrogate models effectively uncovered these pairwise interactions (Figure 3B, Figure S3–S4B).

We also observed that family cluster size has only a minor effect on the outcomes, which is mainly driven by interactions with the social structure parameter (Figure 3B, Figure S3–4B). Since individuals return home each day, they are more likely to interact with others within their home location and family cluster (as long as the social structure parameter is > 0). Family cluster size determines how many individuals, on average, live within each family cluster, and in combination with social structure, it influences the extent of interaction within those family clusters, thus having some effect on the investigated model outputs.

As we pointed out earlier, the results of our sensitivity analysis depend on the variance present in the system. When some parameters explain a disproportionately large amount of variance in the model output across the entire input domain, the contribution of other parameters can be hard to detect. To address this, one can fix the former parameters at specific values and then conduct a “conditional” sensitivity analysis that only varies the latter parameters to assess their relative effects in this particular subdomain of the parameter space.

**Figure 4.**
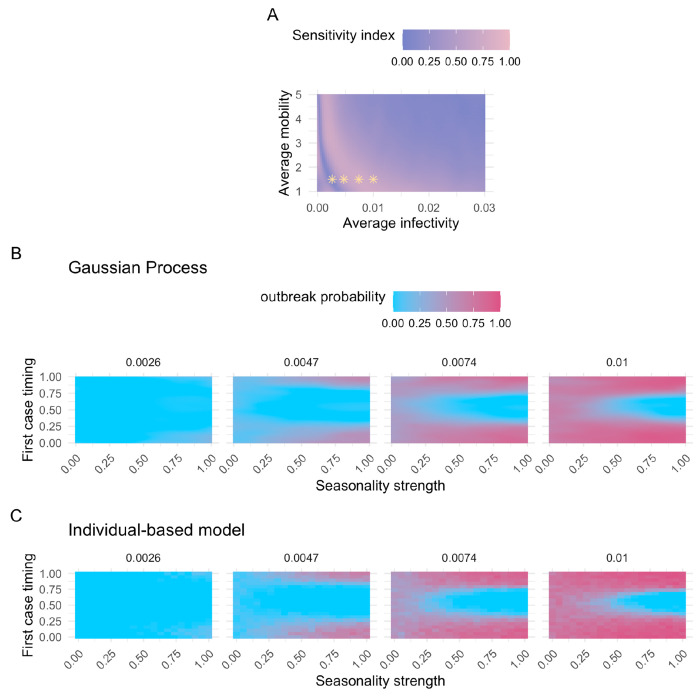
Summary of model outcomes related to outbreak probability. (A) First-order sensitivity index estimates for the first case timing parameter across varying average infectivity and average mobility values. A total of 294,912 points were evaluated for the sensitivity analysis per parameter combination. All other parameters vary across their full ranges (Table 1). The first-order effect measures the influence of a single parameter on the model output (outbreak probability). Yellow stars mark parameter combinations associated with specific model outcomes shown in (B). (B) Predicted outbreak probabilities using the Gaussian Process surrogate model with varying seasonality strength and first case timing values. Panels represent different average infectivities. All other parameters are fixed at default values (Table 1), except for average mobility, which is set to 1.5. (C) Outbreak probabilities inferred from the individual-based model, with varying seasonality strength and first case timing values. Panels represent different average infectivities. As in (B), the remaining parameters are fixed at default values (Table 1), except for average mobility which was set to 1.5. (B) and (C) thus represent model outcomes for the same model parameters, but conducted with the Gaussian Process surrogate model (B) versus the original individual-based model (C), allowing a direct comparison between the two.

We used this approach to analyze the effect of seasonality strength and first case timing on outbreak probability across various average infectivity and average mobility scenarios, since these parameters were previously identified as crucial drivers (Figures 3, S3–4). Interestingly, we observed a sharp increase in the first-order indices for the first case timing when the average infectivity was fixed at a low value, followed by a gradual decline as the average infectivity increased (Figure 4A). This pattern corresponds to a shift in the system: moving from a state where epidemic outbreaks are rare (Figure 4B: 1^st^ panel) to one where outbreaks occur in the majority of simulated scenarios (Figure 4B: 4^th^ panel). Under conditions that are generally unfavorable for disease transmission, outbreaks are rare and occur only when all parameters align to support an outbreak. Consequently, the first-order sensitivity indices for the first case timing parameter tend to be low, because this metric reflects only the independent effects. With increasing average infectivity, the system enters a state where the outbreak probability is primarily driven by the first case timing, creating a hit-or-miss dynamic (Figure 4B: 3^rd^ panel). Here, the strength of seasonality plays a minimal role; the key factor determining whether an outbreak occurs is whether the first case is introduced when infection probabilities are above or below the average infectivity. As average infectivity continues to increase, we observe a U-shape on the outbreak probability heatmap, especially when first case timing coincides with conditions unfavorable for disease transmission (Figure 4B: 4^th^ panel). This reflects how, at this stage, seasonality strength again becomes a critical contributor to outbreak probability. As the average infectivity increases further, the proportion of unlikely outbreak scenarios shrinks, causing the sensitivity index of the first case timing parameter to gradually decrease again. We confirmed that these patterns are not artifacts of the GP model by validating them with the IBM, which showed the same trend (Figure 4C).

### Application to empirical dengue incidence data

To parameterize our model and corroborate its findings with real-world data, we analyzed over a decade of weekly dengue incidence data from Colombia^34^, along with municipality-level processed demographic and environmental data from Siraj *et al.* (2018)^35^. We retrieved weekly dengue incidence data for Colombia from the OpenDengue database^34^, covering January 1^st^, 2007, to December 31^st^, 2019, resulting in 163,279 entries. We selected this cutoff date to avoid confounding effects from the COVID-19 pandemic. To account for potential under-reporting and asymptomatic cases, we adjusted reported dengue incidences by a correction factor of 25^43,44^. We focused on 211 municipalities with populations of at least 30,000 individuals and dengue maximum incidence rates of at least 0.1%. We defined outbreaks as periods of at least four consecutive weeks exceeding the median dengue incidence rate, resulting in the identification of 1,211 epidemic outbreaks.

On this data set, we first tested our model’s prediction of a potential inverse relationship between the average infectivity and average human mobility (Figure S3C). Because we lacked direct fine-scale data, we used mosquito abundance probabilities^35,45^ as a proxy for average infectivity and the inverse of mean travel time as a proxy for average human mobility^35,46^. Mean travel time reflects the average time it takes to reach a settlement with at least 50,000 people (the settlement might not necessarily be within the municipality itself). Shorter travel times suggest more urbanized areas, potentially resulting in greater human mobility. If this relationship holds, we would expect real epidemics to exhibit a positive correlation between mean travel time and mosquito abundance probabilities. However, no significant positive correlation was found, even when restricting the analysis to municipalities with a travel time at the 85th percentile or above (i.e., very rural areas, Spearman’s rank correlation test: *ρ* = 0.1, *S* = 917,546, *p* = 0.085).

We then tested our model’s prediction that the timing of the first infectious case should play a crucial role in shaping outbreak dynamics whenever seasonality is important. However, empirical data only allows us to observe outbreaks that have been measurable in the population. We do not know when or how the first infectious case was introduced, nor do we have information about instances where an infectious case was introduced but did not result in an epidemic. When testing across all observed outbreaks, binned by week, we find that the distribution of epidemics is not uniform throughout the year (Chi-squared test: *χ*^2^ = 117.85, df = 52, *p* < 0.001), which suggests a seasonal effect, and matches our expectations of dengue outbreak dynamics^43,47^.

Next, we used the GP predicting *i*_*max*_ to systematically explore how different parameter combinations in our IBM can effectively recapitulate the empirical dengue incidence data from Colombia. To account for heterogeneity in dengue risk across regions, we incorporated municipality-specific average infectivities while keeping other parameters constant. We analyzed municipalities with at least three epidemic outbreaks, resulting in a dataset of 1,186 epidemics across 173 municipalities. For each municipality, we used 67% of the data for calibration, and withheld 33% for testing. Calibration was done across all municipalities simultaneously. Withholding a portion of the data allowed us to assess the predictive performance of the calibrated GP in estimating *i*_*max*_ for real-world epidemics.

**Figure 5.**
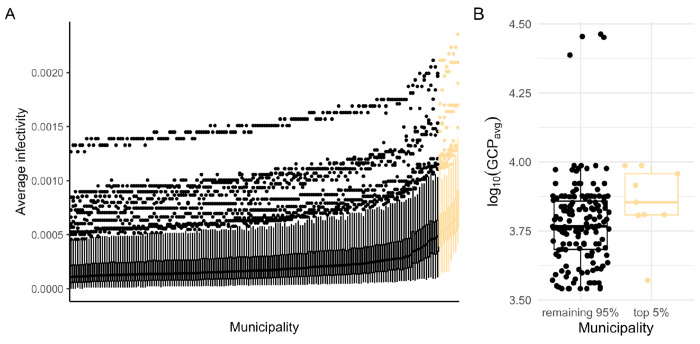
(A) Distribution of municipality-specific average infectivity estimates for the 250 parameter combinations with the lowest root mean square errors. The top 5% of municipalities as sorted by median average infectivity estimates are highlighted in yellow. (B) Average Gross Cell Product (GCP) — a measure of economic activity^66^ where higher values represent greater economic activity — distributions, as reported by Siraj *et al.* (2018), for the municipalities depicted in (A), with the municipalities with the largest average infectivity estimates grouped separately.

The best-fitting parameter combination, which minimized the RMSE on the calibration data, revealed an unexpectedly high degree of social structure in our IBM (seasonality strength = 0.16; first case timing = 0.58; infectious period = 4.67; average mobility = 4.39; mobility skewness = 0.47; social structure = 0.99; family cluster size = 4.54; scaling factor = 0.03). However, across the top 0.1 % (250 out of 25,000) parameter combinations with the lowest RMSE, we observed a wide range of values (Table S1), often spanning the entire parameter range. This suggests that in our IBM, multiple distinct parameter combinations can generate similar epidemic patterns. We also observed variation in average infectivity estimates across municipalities, though certain municipalities consistently exhibited higher average infectivity estimates (Figure 5A). Notably, these municipalities had a significantly higher Gross Cell Product (a measure of economic activity^66^), compared to the remaining municipalities, suggesting a possible link between economic activity and dengue incidence (Figure 5B; *Wilcoxon rank sum test: W* = 1,037, *p* = 0.021). Municipalities with higher average infectivity estimates might merit targeted, in-depth studies to inform public health interventions.

Finally, we evaluated the predictive power of the GP using the best-fit parameters to predict *i*_*max*_ of epidemics across municipalities for the withheld test data. While the model achieved an RMSE of 0.006, the normalized RMSE (RMSE, scaled by the mean of the observed data) was 1.02, indicating that the model struggled to capture the full complexity of the system and that the model’s predictions were not highly accurate (Figure S5A). The rank correlation coefficient between observed and predicted values was 0.458, with permutation tests placing the model in the top percentile, confirming it has some predictive power. However, these results highlight the limitations of our approach when applied to large-scale, heterogeneous epidemic data.

## Discussion

In this paper, we demonstrated the potential of statistical emulation for studying the dynamics of epidemiological IBMs. Specifically, we implemented a dengue-inspired individual-based disease transmission model in C++ and trained Gaussian Process (GP) emulators of that model on three key metrics: outbreak probability, maximum incidence (*i*_*max*_), and outbreak duration. Due to their fast prediction speed, these GPs facilitated highly efficient exploration of the model’s eight-dimensional parameter space, allowing us to conduct comprehensive sensitivity analyses that would otherwise have been computationally prohibitive. Our results show that average infectivity and average mobility have large first-order effects and influence all three epidemiological metrics. The most important pairwise parameter interaction varies by model outcome: the interaction between average infectivity and the average human mobility primarily influences outbreak probability, whereas the timing of the first infectious case, combined with seasonality strength, can shape both *i*_*max*_ and the duration of epidemics. Although our IBM and the trained GPs were not able to capture the full heterogeneity of complex empirical epidemiological data, they still proved able to identify regions in Colombia that might merit targeted public health surveillance and intervention efforts as potential dengue hotspots.

Our IBM’s design is fairly flexible, allowing us to explore a wide range of scenarios, but it nevertheless makes several simplifying assumptions that could limit its accuracy. For example, we modeled an initially completely susceptible population in our simulations, neglecting any preexisting immunities at the onset of the epidemic. This ignores the fact that dengue is caused by four distinct viral serotypes (DENV–1 to DENV–4), and while infection with one strain provides long-lasting immunity against that specific strain, immunity to other strains lasts only a short time^48^. Moreover, a second infection with a different serotype can trigger antibody-dependent enhancement, significantly increasing the risk of severe (and symptomatic) dengue^48^. In hyperendemic countries such as Colombia^43^, where multiple dengue virus serotypes are simultaneously circulating within the population, this can cause complex immunity dynamics. Unfortunately, strain-specific sequencing data and antibody measurements that could be used to accurately estimate the proportion of immune individuals are scarce^43^.

Furthermore, while our IBM incorporates key aspects of dengue epidemiology, such as infectious duration^33^ and the role of human movement^36,40^, we chose not to explicitly model mosquito vectors, which could be important in some scenarios^5^. Combining host models with detailed vector models that account for factors such as habitat availability and selection pressures across mosquito life stages could significantly enhance the realism of epidemiological simulations^2,49^, albeit at a substantial cost in model complexity, number of parameters, and simulation runtime.

Another simplifying assumption in our IBM is in the human mobility model. While the “family cluster size” and “social structure” parameters allow us to model populations with varying levels of social interconnectivity, locations are not spatially explicit, meaning that the distance between them is not defined. Thus, the likelihood of a person visiting a location is solely determined by parameters affecting social population structure and human mobility. Real-world human movement patterns, on the other hand, are known to exhibit strong spatial regularity^8,50,51^. Moreover, in reality, human populations are rarely closed systems like the one we modeled here. Migration and a variety of factors — economic shifts, environmental changes, large public events — often lead to interactions beyond regular social circles, increasing the risk of disease introduction into areas that were previously unaffected^52^.

While we decided to train our GPs on outbreak probability, *i*_*max*_, and duration, a GP could instead be trained on other outputs from the IBM. For example, a GP could be trained on the total epidemic size or the time to the epidemic peak, if relevant to addressing the research question at hand. It would also be possible to use a so-called multi-task GP^53^, which allows the simultaneous prediction of multiple outputs, and is capable of capturing correlations between them. This could improve the efficiency of the training process, especially when the outputs are highly correlated, because multi-task GPs can leverage shared information between the prediction tasks to enhance accuracy and reduce computational costs. Our choice of separate GPs was guided by two factors. First, the outbreak probability GP was trained on the proportion of simulation runs with observed outbreaks, while the *i*_*max*_ and duration GPs were exclusively trained on simulations with observed outbreaks, which made choosing a consistent set of training points across all three metrics challenging. Second, we had no clear expectations regarding the correlation between *i*_*max*_ and outbreak duration: simulations with shorter durations might result from severe epidemics where most individuals are infected rapidly (high *i*_*max*_), or from scenarios where the disease quickly dies out (low *i*_*max*_). These complex dynamics made separate GPs a simpler, more practical choice.

A key factor in implementing a GP is the choice of an appropriate kernel^18,42^. We used the Matérn kernel because of its flexibility in modeling different levels of smoothness in the data. For this kernel, we chose a smoothness parameter *ν* = 0.5, which can be beneficial for capturing model behavior in which small changes in parameters can result in abrupt changes in model outputs, as seen here and in a previous study^16^. Preliminary testing, as well as our trained GPs, showed satisfactory performance with the Matérn kernel, so we did not pursue alternative kernels. Whether the accuracy of our GPs could be improved even further with more customized or composite kernels tailored to specific features of the data remains to be explored.

One key advantage of GPs is their Bayesian nature, which allows for uncertainty quantification. This property is particularly useful in active learning, wherein the uncertainty measurements can be leveraged to choose the most informative points to add to the training data. During GP training, we selected half of the new points based on the confidence interval widths, while the other half was selected using the product of the confidence interval widths and a function of the predicted mean. Specifically, we weighted the confidence interval widths based on how close the predicted mean is to its most extreme possible values, assigning the highest weights to intermediate predictions. This approach encourages the GPs to move away from the edges of the parameter space, where uncertainties are naturally higher and predicted means often become extreme. These extremes occur either due to expected model behavior at the parameter boundaries (extreme parameter values cause extreme model behavior), or because data is sparse in these regions, causing the GP to revert to its prior (a constant mean of 0 in our case, which is an extreme value relative to the average predicted value).^18^. However, this approach might overlook regions that the GP does not determine to be highly uncertain but which could provide valuable information if explored. Alternative sampling strategies, such as expected improvement, could help identify points that boost model performance, even if their initial uncertainty is lower. Moreover, tools like BoTorch^54^ provide libraries to implement advanced batch optimization techniques, allowing the selection of sets of data points that are chosen together to maximize their combined impact on improving GP performance. While more advanced techniques like expected improvement scores and batch optimization could potentially enhance GP performance, they would require further model tuning and validation, which is beyond the scope of this study.

The fast prediction speed of the trained GPs allowed us to conduct comprehensive variance-based sensitivity analyses. However, this approach could be confounded by potential discrepancies between the GP surrogate model and the original IBM. While the GPs generally predicted epidemiological metrics inferred from the IBM well, the width of the sensitivity analysis confidence intervals should be interpreted cautiously. Furthermore, average infectivity and average mobility emerged as the dominant contributors to variance in the epidemiological metrics, making it harder to detect the influence of the other parameters. This scaling effect can obscure smaller, but still relevant, factors. To address this, we also performed sensitivity analyses in targeted regions of the parameter space for which average infectivity and average mobility were fixed, revealing state changes within the model’s dynamics — sudden transitions from rare epidemic outbreaks to frequent outbreaks — which were confirmed by simulating selected points directly with the IBM. However, it is important to note that while a sensitivity analysis captures the variance in model outputs due to parameter changes, it does not fully capture the underlying dynamics of the model, such as state transitions or the mechanistic interactions between single parameters that drive these changes. Specifically, the sensitivity analysis highlights which parameters contribute most to the output variance, but it does not reveal why certain parameter combinations lead to changes in the model behavior.

We observed the largest second-order effects between average infectivity and average mobility for the outbreak probability metric, and between seasonality strength and first case timing for *i_max_* and duration. However, our analysis of over a decade of dengue incidence data from Colombia did not yield the correlation that we expected between our proxies for average human mobility and average infectivity in actual outbreaks. This aligns with previous findings that mean travel time is a broad measure of accessibility rather than a precise indicator of actual human mobility^35^. Furthermore, we used mosquito abundance probabilities^35,45^ as proxies for the average infectivity, implicitly assuming a constant mosquito biting rate such that a higher abundance or likelihood of mosquitoes corresponds to an increased disease transmission risk. However, in reality, this relationship is more complex. Mosquito abundances can be significantly influenced by human alterations to the environment, such as vector control measures or urbanization^55^. Additionally, infection risk depends on other factors, such as human-mosquito contact rates^56^ and the behavior of individual mosquitoes^57^. While our analysis suggests — in line with previous studies^43,47^ — a seasonal distribution of dengue epidemics, we assumed that each epidemic outbreak is independent. This means that we did not account for potential correlations between outbreaks occurring within the same municipality over time, or spatial correlations across neighboring municipalities. As a result, the actual *p*-value of our test should be interpreted with caution.

When comparing the predictions of our model with real-world outbreaks, the only empirical parameter that actually varied within each municipality was the timing of the epidemic’s onset. This limited the GP’s flexibility to generate diverse predictions. In fact, a simple linear mixed-effects model that predicts log_10_-transformed *i_max_* values based on the onset timing of an epidemic, while accounting for the municipality-level variations with random effects, performed similarly to the GP model on withheld test data (Spearman’s *ρ* = 0.54). This suggests that both the abstract IBM and the GP emulators might be too generalized to effectively predict real-world outbreak data across multiple municipalities. To achieve more accurate predictions, the IBM would need to be more complex, incorporating municipality-specific characteristics such as outbreak histories, population immunity levels, and finer-scale human movement patterns, which might be critical for capturing the nuanced dynamics of local outbreaks.

Despite the GP struggling to predict complex empirical data, we observed that a subset of municipalities consistently had higher average infectivity estimates than the other municipalities. Several of these outlier municipalities are notable for their economic or geographic importance. For example, Puerto López, which had the highest average infectivity estimate, is a key river port, while Leticia, another outlier, is located at the intersection of the borders of Colombia, Brazil, and Peru, serving as a major port on the Amazon River. Tourist destinations like Melgar and La Mesa also exhibited elevated average infectivity estimates, possibly because of increased connectivity due to tourism and travel, which might contribute to higher dengue transmission rates in these areas. This observation supports the idea that human movement and economic activity could play a significant role in shaping dengue dynamics in these municipalities^58^. Furthermore, the higher economic activity (Gross Cell Product) in these regions might indicate that a larger proportion of the population has access to healthcare, which could affect our model’s assumptions. Specifically, better healthcare access could lead to higher detection and reporting of dengue cases, thereby violating our assumption of constant reporting rates across municipalities.

In conclusion, we explored the utility of statistical emulation to efficiently analyze epidemiological IBMs. The use of GPs allowed valuable insights into the key drivers of our simulated disease dynamics, revealing critical interactions between average infectivity, human mobility, and seasonality. When analyzing empirical dengue outbreaks, our calibrated GP highlighted municipalities that could serve as candidates for targeted interventions or in-depth studies. Our work demonstrates both the potential and the challenges of using statistical emulation to explore complex epidemiological systems, providing a foundation for future efforts that could incorporate additional model complexity and realism while maintaining computational efficiency.

## Methods

### Individual-based model

This detailed individual-based model (IBM) description is motivated by previous studies using the ODD protocol for describing IBMs^1,5,59^. The IBM was implemented in C++.

#### 1. Purpose and patterns

The purpose of our IBM is to explore how human movement, social population structure, and seasonal variation in infection probability influence infectious disease dynamics. Rather than replicating a specific empirical system, the model is intended to highlight the relative importance of these parameters and their interactions in shaping disease spread. Its performance is evaluated by examining how the absolute number of infectious cases and the change in infectious cases over time are affected by IBM parameter changes. The model is abstract, but is designed to loosely replicate dengue transmission dynamics.

#### 2. Entities, state variables, and scales

The model operates in daily timesteps and includes two primary entities: humans and locations. Each location represents a residential home with a group of residents, who are collectively considered a ‘family’. There are no distinct non-home locations such as workplaces, schools, or other public spaces. The number of humans in a given family — the number of humans residing at a given home location — is drawn from a negative binomial distribution with *μ* = 6.2 *and θ* = 9.07, following the household size distribution observed in Iquitos, Peru^36,37^.

Each individual human has the following state variables: a home location, an infection status (susceptible, exposed, infected, or recovered), the number of remaining days in their current infection status (how much longer they will stay in their current state), and the number of elapsed days in their current infection status (how long they have already been in that state).

The basic spatial unit of the model is the location. Simulations are initialized by generating 10,000 locations. Each location has the following state variables: the number of infectious humans visiting it in the current timestep of the model, its per-contact infection probability (the probability that an infectious individual that visited the location at time *t-1* transmits the disease to a susceptible individual that visits the same location at time *t*), a history of the number of infectious visitors it has had, and a history of its infection probability over time. All locations are randomly grouped into family clusters of a user-specified size. Each location is assigned to only one family cluster, ensuring that no locations are shared between clusters. All family members of a single home location belong to the same family cluster, but not all members of a given family cluster reside in the same home location. Figure S1 provides a conceptual overview of family clusters (and human movement) as implemented in our IBM Conceptually then, a family cluster is a group of locations, the members of which tend to socialize together; an individual in a given family cluster is more likely to visit a location inside the family cluster than to visit a location outside it. This grouping introduces social structure into the simulation (please refer to the next section for further details). Note that “family clusters” are conceptually equivalent to the “social groups” defined by Reiner *et al.* (2014)^37^.

#### 3. Process overview and scheduling

The two core processes of the model are human movement and infection dynamics.

##### Human movement

For the process of human movement, during each timestep the model iterates through all individuals to determine which locations they will visit. Each individual visits its home location at least once every timestep. For each individual, an additional number of visits by the individual is drawn from a negative binomial distribution at the beginning of each day. The use of a negative binomial distribution allows for heavy-tailed human mobility distributions where some individuals are highly mobile, visiting a large number of locations per day. However, there is no correlation between the various sampled values for a given individual over time, meaning that we did not model systematic super-spreaders in our IBM. Given that a large fraction of empirical dengue infections is asymptomatic^60^, the infection status does not affect human movement in our IBM: infectious individuals visit, on average, the same number of locations as susceptible or recovered individuals.

For each visit, whether the visit is to a location inside the individual’s family cluster or to a location outside that cluster is determined probabilistically, with the social structure parameter being the probability that a particular visit happens within the family cluster (Table 1). Locations to be visited are then randomly selected from the set of locations inside or outside the individual’s family cluster, as appropriate. Multiple visits to the same location are allowed. Figure S1 provides a conceptual overview of human movement patterns as implemented in our IBM.

##### Infection dynamics

In our model, each human can have one of four infectious states: susceptible, exposed, infectious, and recovered. The model tracks the number of days each simulated human will remain in its current infection state, decreasing this count at the end of each day.

At the start of the simulation, all humans are susceptible. The disease is then introduced into the population by randomly selecting one individual and immediately changing their infection status from susceptible to exposed. This exposed status indicates that the individual has contracted the disease but is not yet infectious. Unless specified otherwise, exposed individuals become infectious at the end of each day, effectively reducing the model to a Susceptible-Infectious-Recovered model, where the length of the infectious period is specified by the user. At the end of an individual’s infectious period, the individual’s infection status changes to recovered the next day. The model assumes lasting immunity, so once individuals recover, they cannot be reinfected.

If a susceptible human visits a location that had *N* infectious visitors the day before, the probability of contracting the disease and immediately entering the exposed state is 1 – (1 – *p_infection_*)*^N^* where *p_infection_* is the infection probability per contact. (This is simply the probability that a binomial draw B(*N*, *p_infection_*(*t*)) ≥ 1, indicating that infection occurred from at least one previous visitor.) The infection probability *p_infection_* follows a cosine function that is determined by three parameters: the average infectivity (α_0_), the seasonality strength (α*_season_*), and the first case timing (*t_first_*), and is calculated as follows:

$$p_{\text{infection}}(t)={\alpha_{0}}^{*}\left(1+\alpha_{\text{season}}*\cos\left(2\pi *\left(t/365-t_{\text{first}}\right)\right)\right).$$

Each location is assigned the same infection probability for a given day, as determined by the cosine function above. Variations in the overall likelihood of infection, from location to location, arise from the differing numbers of infectious individuals visiting each location.

If a visiting human is already infectious, the model increments the count of infectious visits for the current day at each location visited by that human, which will make those locations infectious in the following timestep as just described.

Since humans only change their infection status at the end of each day, and the likelihood of infection for susceptible individuals is determined by the number of infectious visitors from the previous day, the order in which individuals are processed is inconsequential. This ensures that the model remains asynchronous and order-independent during each day. Indeed, this design would allow the model to be parallelized to run across multiple processing cores, although runtimes were fast enough that we did not deem that necessary.

#### 4. Design concepts

##### Basic principles

The model aims to study abstract disease dynamics within human populations exhibiting varying levels of social structure. It does not focus on the realistic modeling of a specific city, or on the biological details of dengue transmission dynamics.

##### Emergence

The number of infectious individuals each day — the central output of this model, for our purposes — is an emergent property, not predefined within the model. Stochasticity plays a major role in introducing uncertainty into these patterns.

##### Adaptation, objectives, learning, prediction, and sensing

None of the individuals in the model have the ability to adjust their behaviors. There are no adaptive behaviors, learning abilities, predictive capabilities, or sensing capabilities in the model.

##### Interaction

Humans interact by potentially infecting other humans who visit the same location the next day.

##### Stochasticity

This IBM incorporates stochasticity in the family sizes, the daily number of visits per human, and the probabilistic infection dynamics. For detailed descriptions, please refer to Sections 2 and 3.

##### Collectives

Each human is assigned a home location and a family cluster, making them members of a family and a collection of locations. However, no special properties are attributed to sharing a home location or family cluster, except for the general tendency to interact more frequently due to the human movement rules described in Section 3.

##### Observations

The model outputs a table of the counts of susceptible, exposed, infectious, and recovered individuals for each day of the simulation, across the entire population.

#### 5. Initialization

Please refer to Sections 2 and 3 regarding the initialization of the model.

#### 6. Input

The model has eight parameters that can be specified by the user with command-line arguments (Table 1).

Out of these eight parameters, three parameters collectively influence the infection probability, as described by the equation in Section 3: the average infectivity, the seasonality strength, and the first case timing. Additionally, the user must define:

- The length of the infectious period, after which infectious individuals transition to being recovered (Section 3: *infection dynamics*).
- Parameters for the negative binomial distribution describing human movement (Section 3: *human movement*).
- The proportion of visits that occur within the family cluster of an individual (Section 3: *human movement*).
- The number of locations per family cluster (Section 2).

### Gaussian Processes

Statistical emulation involves replacing an individual-based simulation framework with a statistical surrogate model, or machine learning model trained on input-output pairs from the original framework^12^. For this task, we used GPs, which are non-parametric models that define a distribution over functions. This allows GPs to efficiently interpolate between scarcely sampled observations. GPs are an attractive choice, not only due to their mathematical tractability, but also because their Bayesian nature allows for the quantification of model uncertainty. We incorporated these uncertainty estimates into our policy for scoring potential additional data points (Figure 1A). We implemented GPs in Python (v3.10.6) using the GPyTorch library (v1.11)^19^ for efficient GP modeling, and the torch.cuda module from the PyTorch package (v2.0.1)^61^ for GPU acceleration with NVIDIA GPUs. The implementation was inspired by a GP surrogate model previously used to study the efficiency of gene drives in rat populations^16^.

We trained a separate GP model for each of the three key characteristics described in the previous section: outbreak probability (proportion of simulation runs in which more than 0.1% of the population becomes infected), *i_max_* (highest proportion of infectious individuals at any day) and epidemic duration (timespan from the first infectious case to the recovery of the last infectious individual). While the IBM outputs for outbreak probability and *i_max_* are bounded between 0 and 1, epidemic duration spans a much wider range: In the initial training dataset (N = 5,000 data points), the observed duration of epidemics ranged from 19.65 to 424.15 days. To manage the variance in epidemic duration and improve the GP’s ability to predict longer epidemics, we applied a logarithmic transformation to the outbreak duration.

The response variable for each GP is calculated from 100 simulation runs per parameter combination. While outbreak probability is defined as the proportion of simulation runs with observed outbreaks, *i_max_* and outbreak duration are exclusively calculated from simulations with epidemic outbreaks. This approach helps minimize unrepresentative input-output pairs. For example, if a specific parameter combination results in an *i_max_* of 10%, but only 50% of the simulation runs result in an outbreak, averaging across all simulations would result in an *i_max_* estimate of about 5%, not accurately representing the dynamics of the IBM.

The covariance function — or kernel — of a GP determines how much the response values of different input points covary^42^. Thus, the choice of kernel is crucial in shaping the GP’s predictions. We selected a Matérn kernel with *ν* = 0.5, which corresponds to the exponential kernel. This kernel is capable of capturing abrupt changes in function values^18^. We applied the same kernel type across all three GPs.

### Gaussian Process training loop

One key advantage of GPs over other machine learning methods is their Bayesian nature, which allows us to identify regions in the parameter space where the model’s predictions are highly uncertain. This allows us to strategically select new training data from areas where the GP is least confident, thereby enhancing prediction accuracy. This approach forms the basis of the GP’s active learning loop (Figure 1A), which consists of three steps: training the GP, scoring potential new training data, and then updating the training data with selected new data points^18^.

#### Step 1: GP training

We trained each GP for 16 rounds: one initial round with a training dataset consisting of a Latin hypercube sample (LHS) of 5,000 data points from the entire input domain (Table 1), followed by 15 active training rounds (Figure 1A). For training, we utilized the Adam optimizer from PyTorch^61^ with a learning rate of 0.01. In each training round, the GP was trained for 30,000 iterations, with a model snapshot saved every 1,000 iterations. To avoid overfitting, we evaluated all 30 snapshots against a separate validation dataset consisting of 10,000 LHS points. We selected the snapshot with the lowest RMSE on the validation dataset for step 2 in the training loop.

#### Step 2: Data scoring

In each active learning round, we scored 10^7^ LHS points using two distinct policies^16^. These scores are used as probability weights to select 1,000 new data points to expand the training data. Policy 1 is based solely on model uncertainty. In this policy, the probability *p_i_* that a data point *i* is selected is proportional to the width of the 95% confidence interval for that point (*w_i_*), normalized by the total width of all potential data points:

$$p_{i}=\frac{w_{i}}{\sum w}$$

Policy 1 assigns larger weights to data points with greater uncertainties. However, regions with large uncertainties are often clustered near the edges of the observed parameter space, where the GP must extrapolate far beyond observed training data^42^. However, while the uncertainty bounds of these points might be relatively high, the degree of improvement the GP can gain from sampling points from the edges of the parameter space can be limited. To avoid oversampling these areas, we developed policy 2. Policy 2 reduces the likelihood of sampling points with extreme predicted values. Specifically, the 95% confidence intervals from policy 1 are further weighted by the GP’s prediction. The probability *p_i_* of selecting a data point *i* is given by:

$$p_{i}=\frac{w_{i}\cdot \left(\left(m_{\max}-m_{i}\right)\cdot m_{i}+1/n\right)}{\sum w\cdot \left(\left(m_{\max}-m\right)\cdot m+1/n\right)}$$

where:

- *w_i_* is the 95% confidence interval width for point *i*
- *m_i_* is the GP’s predicted value for point *i*
- *m_max_* is the maximum predicted value (3 for duration, 1 otherwise)
- *n* is the total number of potential data points

This formulation ensures that points with high uncertainty yet with predicted values near the midpoint of the range are assigned the highest weights. The GP’s predictions were clipped to the range [0, 1] for outbreak probability and *i_max_*, and to [0, 3] for epidemic duration (since the GP predicts log_10_-transformed durations, this range corresponds to durations between 1 and 1,000 days).

For our adaptive sampling strategy, 50% of the points are selected using policy 1, and the remaining 50% are selected using policy 2.

#### Step 3: Update training data

As mentioned earlier, the data points for *i_max_* and duration are based solely on simulation runs where epidemic outbreaks occurred. If a selected data point did not result in 100 outbreaks after 2,000 simulation attempts, we chose a new data point. For the initial training dataset, where no GP predictions were available, this selection was done randomly from 10^7^ LHS samples. In the active learning rounds, we chose all of the new data points as described in step 2. After successfully simulating all selected points, the new results are added to the training dataset, and a new GP training cycle begins (Figure 1A).

### Gaussian Process usage

We evaluated the accuracy of the trained GP using an independent test dataset of 10,000 LHS points and calculated the RMSE. For visualization purposes, such as in heatmaps (e.g., Figure 3C), we clipped the predictions to the range [0, 1] for epidemic probability and *i_max_*, and to [0, 3] for the log_10_-transformed epidemic duration.

### Sensitivity analysis

To explore how changes in parameters affect the epidemic metrics, we conducted a series of variance-based sensitivity analyses^62^ in Python using the Sobol method implemented in the SALib library (v1.4.7)^63^. We performed sensitivity analyses on the output of GP models rather than the IBM due to the GP’s faster prediction capabilities.

The Sobol method quantifies the contribution of single parameters and their interactions to the variance of a model’s output. Since these variance components are often not analytically tractable, the Sobol method approximates them using a Monte Carlo method. The resulting sensitivity indices — first, second, and total order — provide a measure of each parameter’s influence. First-order effects measure single parameter contribution, second-order effects measure the interactions of two parameters, and total order effects capture the combined impact of each parameter, including all interactions with other parameters of any order.

To perform the sensitivity analysis, the number of model evaluations is proportional to *n* * (*2d* + *2*), where *n* is the base sample size and *d* is the dimensionality of the parameter space (*d* = 8; Table 1)^63^. The accuracy of the Sobol indices improves with a larger *n*, leading to smaller confidence intervals. For the sensitivity analysis of the entire input domain, where all parameters vary across their full range (Table 1, Figure 3A–B), we selected *n* = 2^19^. To investigate the first-order effect of the first case timing with the two most influential parameters (average infectivity and average mobility) held constant, we conducted a sensitivity analysis with *n* = 2^14^ for each combination of these parameters (Figure 4A). We calculated 95% confidence intervals of the sobol indices using the bootstrapping method provided by SALib^63^.

### Empirical data

We retrieved weekly dengue incidence data at the municipality level for Colombia from the OpenDengue database, an open-access platform that provides detailed epidemiological data on dengue^34^. The selected dataset spans from January 1^st^, 2007, when weekly resolution data became consistently available, to December 31^st^, 2019, comprising 163,279 entries. We chose to end in 2019 to avoid the potential confounding effects of the COVID-19 pandemic^64^. We chose Colombia for this study because it is one of the countries most affected by dengue in the Americas^43^ and offers exceptionally well-documented time series data on dengue incidence^34^. To adjust dengue incidences for potential under-reporting and asymptomatic cases, reported dengue incidences were corrected by a factor of 25^43,44^. Estimates for under-reporting factors in dengue typically range from 10 to 27, depending on the region and study^44^. While we recognize that using a correction factor of 25 for Colombia, which has well-documented dengue incidence records^34^, might be cautious, we chose it to include a broad range of municipalities.

We obtained municipality-level processed data from Siraj *et al.* (2018), which provides a global, high-resolution dataset of potential environmental drivers for Zika transmission in Colombia between January 1^st^, 2014 and October 1^st^, 2016. Although the data published by Siraj *et al.* (2018) focused on Zika, it is relevant to dengue because both viruses share a primary vector, *Ae. aegypti*, which is responsible for the majority of dengue transmission in Colombia^65^. Specifically, we used four metrics from Siraj *et. al* (2018) (i) the population count (ii) the weekly occurrence probabilities of *Ae. aegypti* ^45^(iii) the Gross Cell Product, which measures economic activity at a fine spatial scale^66^, and (iv) the mean travel time to the nearest city. Please refer to Table 1 in Siraj *et al.* (2018) for additional information on the municipality-specific data.

We matched the records from Siraj *et al.* (2018) and Clarke *et al.* (2024) based on the names of municipalities and their respective departments. To improve the matching, we standardized the municipality and department names by converting them to lowercase and applying a latin-ascii transformation to remove any accents or special characters. In cases where mismatches occurred, either at the municipality or departmental level, we followed a similar approach to Clarke *et al.* (2024), manually reviewing the records and checking the geographic boundaries using shapefiles. While we were able to obtain the original shapefiles from Clarke *et al.* (2024) (Oliver Brady, personal communication), the original shapefiles for Siraj *et al.* (2018) were not accessible at the time of our study. As a substitute, we used shapefiles from the OCHA database^67^. Despite this limitation, we successfully matched 95% (1,009 out of 1,063) of the municipalities present in the raw dengue incidence data. Our goal was not to achieve a perfect match, but rather to secure a sufficient number of high-quality matches to proceed with our analysis.

We focused on 211 municipalities that aligned with our IBM in terms of population size (i.e., at least 30,000 individuals) and had a maximum dengue incidence rate of at least 0.1% over the entire study period. To detect epidemics, we fitted a smoothing spline using the ss() function from the npreg R-package (λ =10^−10^)^68^. An epidemic outbreak was defined as a period of at least four consecutive weeks in which the spline function exceeded the median dengue incidence rate. Using this method, we identified 1,211 potential epidemic outbreaks with an *i_max_* of at least 0.1% which were included in the analysis. On average, each municipality had 6.34 outbreaks. The average duration per outbreak was 195 days, with an average *i_max_* of 0.6%.

### Parameter exploration with Gaussian Processes

The speed of the GP predictions allows for an efficient exploration of which parameter combinations in our epidemiological IBM provide a best fit to the dengue incidence data from Colombia. This also allows us to assess whether our GPs — and consequently, our IBM — can provide insights into real-world epidemic outbreaks. To capture the heterogeneity in dengue transmission potential, we incorporated municipality-specific average infectivities while assuming that other model parameters remained constant across all municipalities. We focused on municipalities with at least three outbreaks, resulting in a total of 1,186 epidemics across 173 municipalities. For each municipality, we randomly split the data into 67% for parameter fitting (N = 737) and 33% for testing (N = 449), using the test data to evaluate the GP’s predictive performance for *i_max_*.

We generated 25,000 LHS parameter combinations from the full parameter space (Table 1), excluding the average infectivity parameter. Additionally, we introduced a scaling parameter (ranging from 0 to 0.1) to adjust for discrepancies between the simulated and observed dengue incidence rates, which might arise due to the abstraction in our model as compared to real-world data. This scaling factor applies uniformly to all incidence values, while preserving the relative differences between single outbreaks. For each of the 25,000 LHS samples, we tested 50 evenly spaced average infectivities over the range [0, 0.03], resulting in 1.25 million predicted data points for the 737 epidemics used for calibration. For each epidemic, we accounted for the epidemic’s start time by adjusting the first case timing parameter, while using municipality-specific infection probabilities. The predictions were constrained to the range [0, 1] before calculating the RMSE between observed and predicted *i_max_*. We then selected the average infectivity that minimized the RMSE for each of the 25,000 LHS in each municipality. The 25,000 parameter combinations were ranked by summing the RMSE across all municipalities, using the best-fit (i.e., lowest RMSE) infection probabilities for each. To further investigate infection probabilities across municipalities, we examined the 250 LHS combinations with the lowest RMSE sums.

Finally, we evaluated the GP’s predictive performance on the withheld test data by calculating both the RMSE and Spearman’s rank correlation coefficient (*ρ*). To test the significance of Spearman’s *ρ*, we conducted 1,000 permutation tests, where the start time and municipality for each epidemic in the test set were randomly shuffled.

### Statistical analysis

Unless stated otherwise, statistical analyses were performed using the R statistical computing environment (v4.2.1)^69^. Significance is declared at an alpha cut-off of 5%.

### Code availability

The source code of the individual-based disease transmission model implemented in C++ is available on GitHub at https://github.com/AnnaMariaL/DengueSim. Simulated data, pre-trained GPs, and Jupyter notebooks demonstrating the usage of the GPs are also available on GitHub at https://github.com/AnnaMariaL/DengueSim-GP.

## Supplementary Material

1

## Figures and Tables

**Table 1.** Parameters of the individual-based disease transmission model

Parameter	Description	Default	Range
Average infectivity	The average infection probability across a year, which removes the effect of seasonality	0.015	[0, 0.03]
Seasonality strength	A scaling factor between 0 and 1 that controls the magnitude of seasonal variation in infection probability	0.5	[0, 1]
First case timing	Determines the timing of the first case relative to the seasonal peak in infection probability	0	[0, 1]
Infectious period	The average number of days an individual remains infectious, with actual days determined by probabilistically rounding to the nearest integers around the specified value	5	[4, 6]
Average mobility	The average number of visits a human makes to locations per day, in addition to their family home	2	[1, 5]
Mobility skewness	The success probability in the negative binomial distribution that determines the number of visits a human makes per day; a lower value results in greater variance in the daily visit count	0.5	[0.05, 0.95]
Social structure	The probability of a visit occurring within the family cluster of the individual moving	0.5	[0, 1]
Family cluster size	The average number of locations assigned to each cluster, with actual sizes determined by probabilistically rounding to the nearest integers around the specified value	5	[1, 20]

## Glossary

GP: Gaussian Process
IBM: Individual-based model
RMSE: Root mean square error
LHS: Latin hypercube sample
*i_max_*: Maximum incidence
