# Supplementary material for: Gaussian Process Emulation for Modeling Dengue Outbreak Dynamics
Source: medRxiv. 2024 Nov 29:2024.11.28.24318136. Preprint. [Version 1] doi: 10.1101/2024.11.28.24318136 (PMC11623728; doi:10.1101/2024.11.28.24318136)
Supplement: Supplementary file 1 [file NIHPP2024.11.28.24318136v1-supplement-1.pdf]

medRxiv preprint doi: https://doi.org/10.1101/2024.11.28.24318136; this version posted November 29, 2024. The copyright holder for this preprint (which was not certified by peer review) is the author/funder, who has granted medRxiv a license to display the preprint in perpetuity.
It is made available under a CC-BY 4.0 International license .

Supplementary information

Supplementary figures

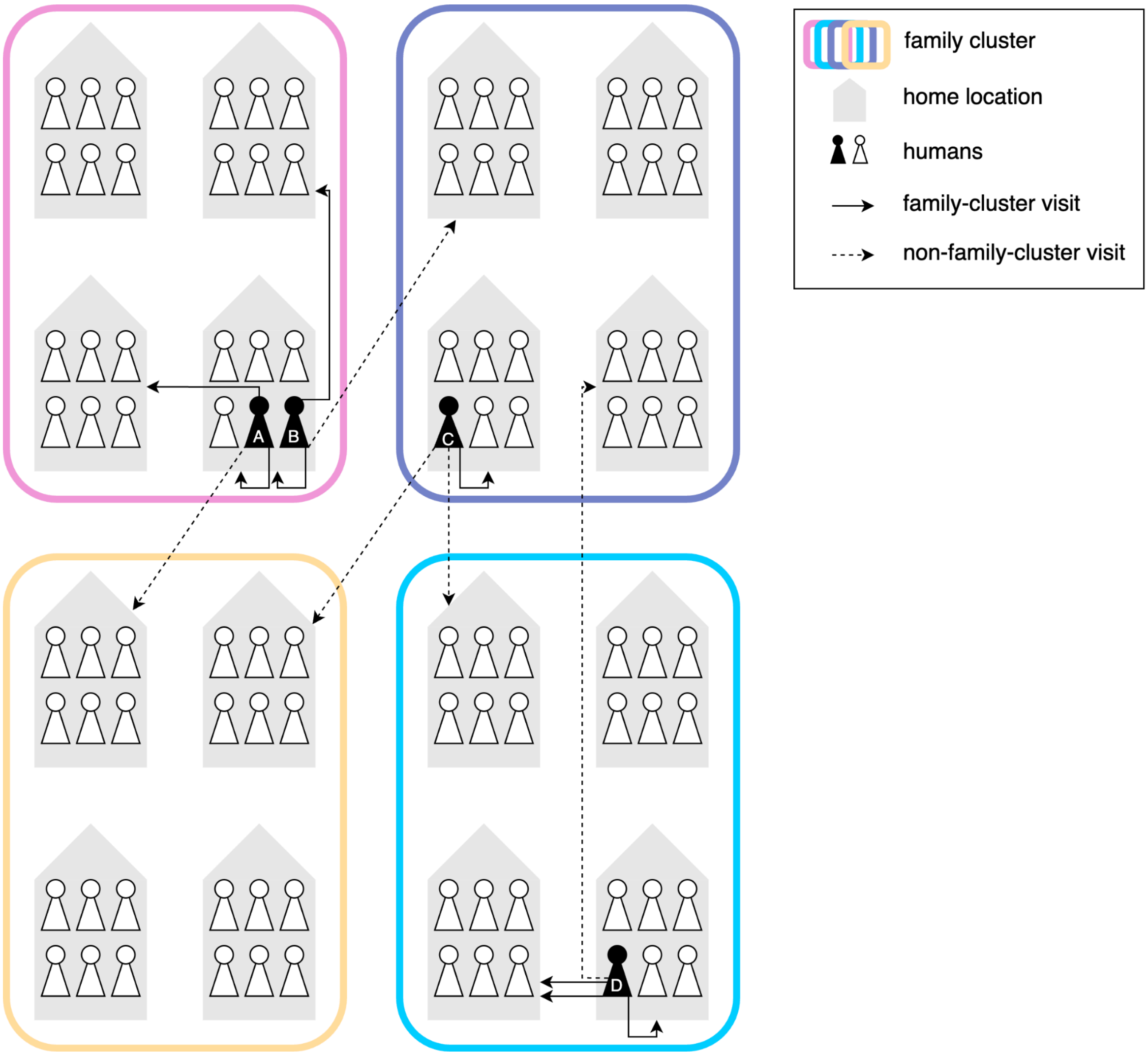

Figure S1. Schematic overview of human movement in the individual-based model. Each colored frame represents a unique, non-overlapping family cluster, with each cluster containing multiple family homes. Individuals can make visits within their own family cluster (solid arrows) or to other clusters (dashed arrows). The likelihood of visits occurring inside the family cluster is determined by the social structure parameter (Table 1). Each individual visits their home at least once per day and moves independently of others in the same family (individuals A and B). Multiple visits to the same location are allowed (individual D). Visits to other family clusters occur randomly and are not restricted to any specific cluster (individual C).

medRxiv preprint doi: https://doi.org/10.1101/2024.11.28.24318136; this version posted November 29, 2024. The copyright holder for this preprint (which was not certified by peer review) is the author/funder, who has granted medRxiv a license to display the preprint in perpetuity.
It is made available under a CC-BY 4.0 International license .

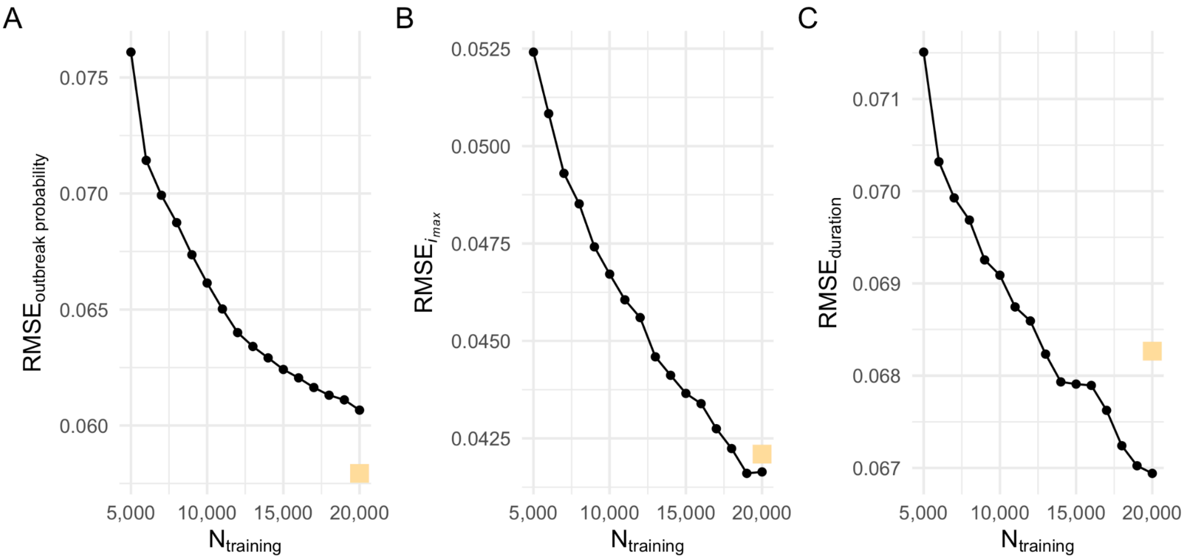

Figure S2. The Root Mean Square Error (RMSE) between the Gaussian Process predictions and the individual-based model results in the validation dataset (N = 10,000 data points). The RMSE decreased as the size of the dataset used to train the Gaussian Processes increased (x-axis). The RMSE between the predictions of the final GP model and the test data (N = 10,000 data points) is indicated by a yellow square. (A) outbreak probability (B) maximum incidence (i_{max}), (C) log_{10}-transformed duration.

medRxiv preprint doi: https://doi.org/10.1101/2024.11.28.24318136; this version posted November 29, 2024. The copyright holder for this preprint (which was not certified by peer review) is the author/funder, who has granted medRxiv a license to display the preprint in perpetuity.
It is made available under a CC-BY 4.0 International license .

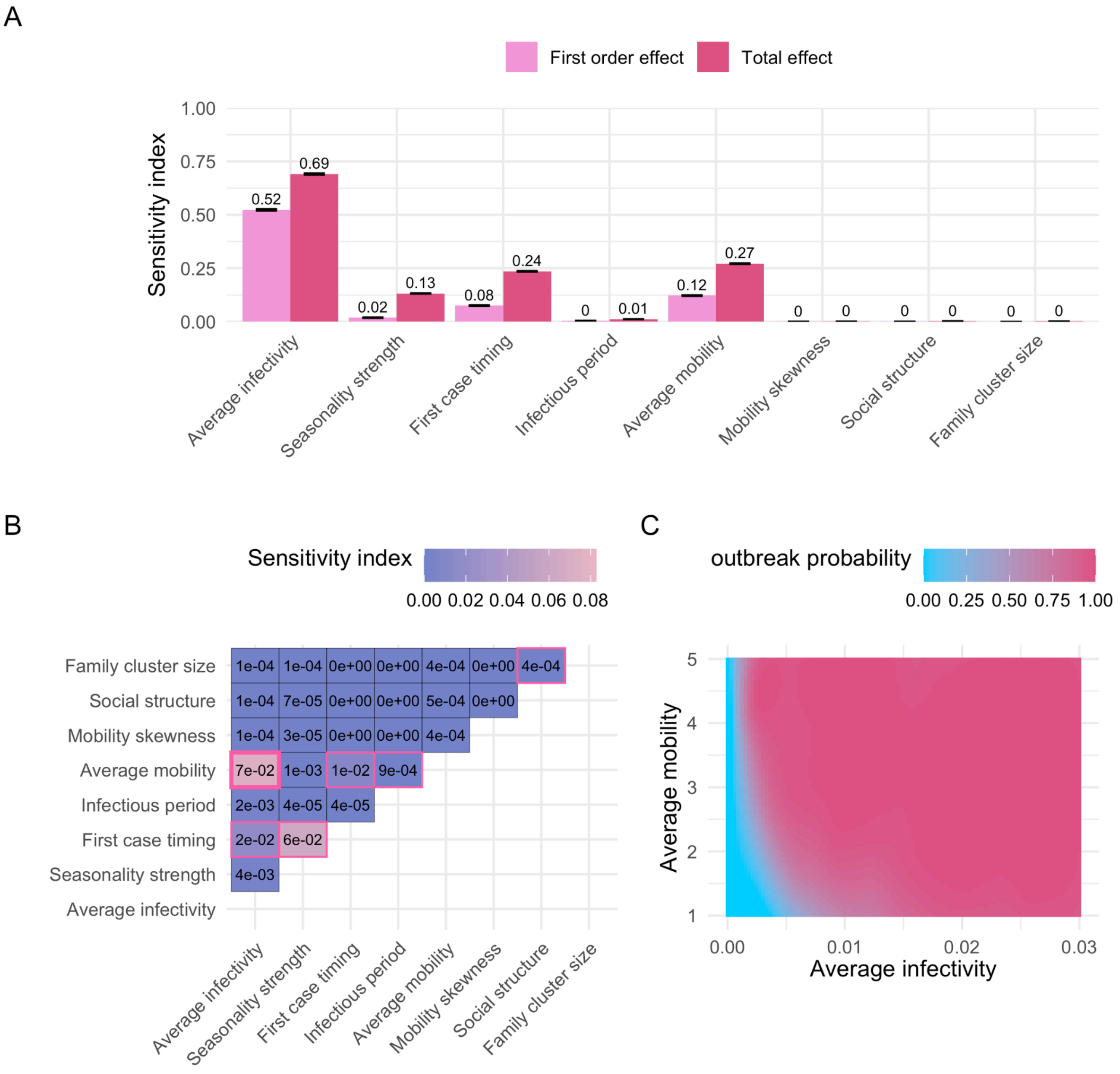

Figure S3. Sobol sensitivity analysis, outbreak probability. (A) First-order and total effects across the entire input domain (Table 1). The first-order effect describes the impact of a single parameter on the model output (outbreak probability), while the total effect accounts for all interactions involving one or more parameters. Error bars represent the 95% confidence intervals of the sensitivity index estimates. A total of 9,437,184 points were evaluated for the sensitivity analysis. (B) Second-order effects across the entire input domain (Table 1). A second-order effect captures the pairwise interaction between two variables. Sobol indices with a 95% confidence interval that does not overlap zero are highlighted with a pink border. The largest second-order effect is emphasized with a bold pink border. (C) Predicted outbreak probabilities with varying "average infectivity" and "average mobility" parameters (i.e., the two parameters with the largest second-order effect, see panel B). Other parameters were fixed at default values (Table 1).

medRxiv preprint doi: https://doi.org/10.1101/2024.11.28.24318136; this version posted November 29, 2024. The copyright holder for this preprint (which was not certified by peer review) is the author/funder, who has granted medRxiv a license to display the preprint in perpetuity.
It is made available under a CC-BY 4.0 International license .

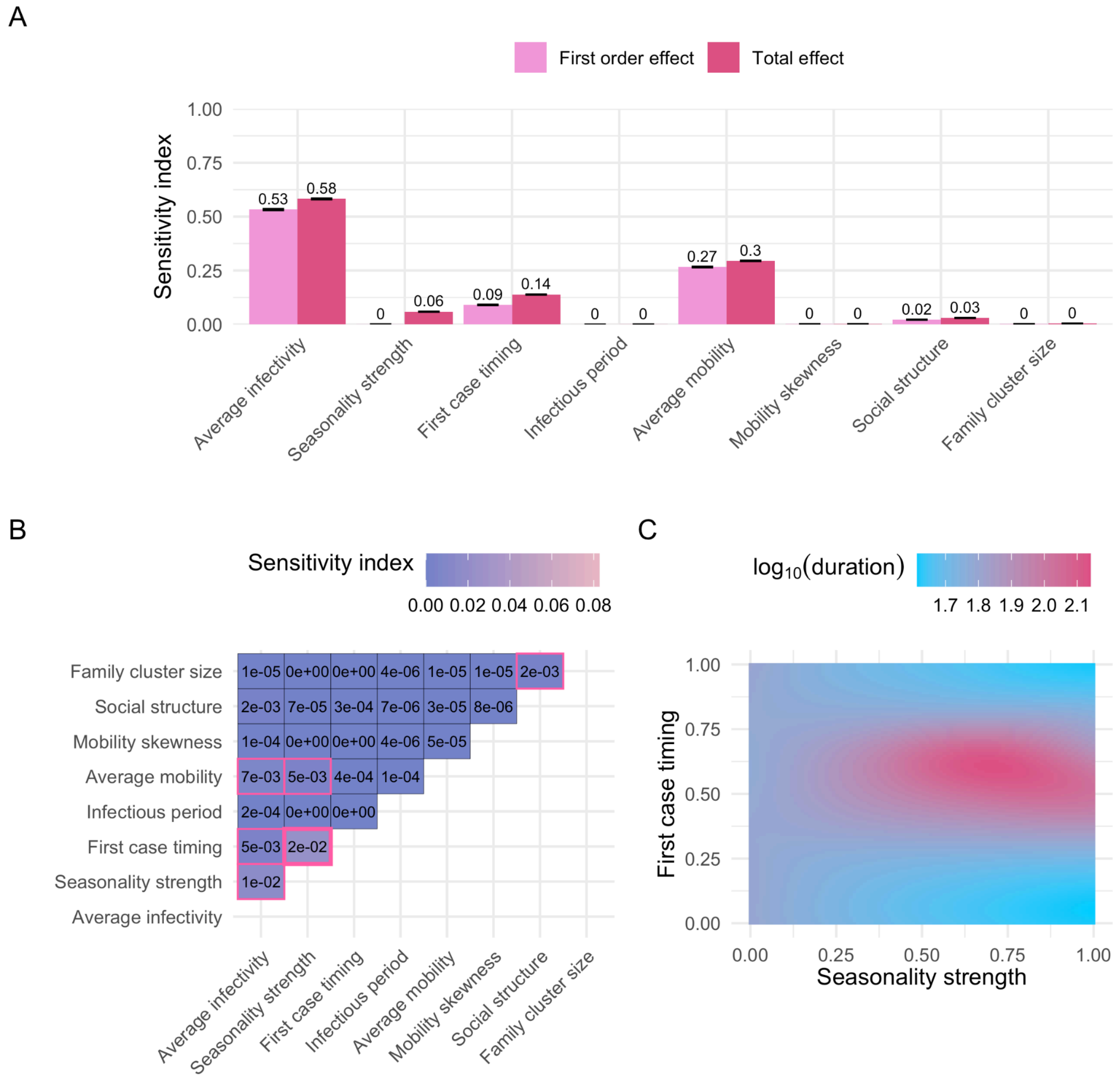

Figure S4. Sobol sensitivity analysis, $\log_{10}$-transformed duration. (A) First-order and total effects across the entire input domain (Table 1). The first-order effect describes the impact of a single parameter on the model output ($\log_{10}$(duration)), while the total effect accounts for all interactions involving one or more parameters. Error bars represent the 95% confidence intervals of the sensitivity index estimates. A total of 9,437,184 points were evaluated for the sensitivity analysis. (B) Second-order effects across the entire input domain (Table 1). A second-order effect captures the pairwise interaction between two variables. Sobol indices with a 95% confidence interval that does not overlap zero are highlighted with a pink border. The largest second-order effect is emphasized with a bold pink border. (C) $\log_{10}$(duration) predictions with varying "seasonality strength" and "first case timing" parameters (i.e., the two parameters with the largest second-order effect, see panel B). Other parameters were fixed at default values (Table 1).

medRxiv preprint doi: https://doi.org/10.1101/2024.11.28.24318136; this version posted November 29, 2024. The copyright holder for this preprint (which was not certified by peer review) is the author/funder, who has granted medRxiv a license to display the preprint in perpetuity.
It is made available under a CC-BY 4.0 International license .

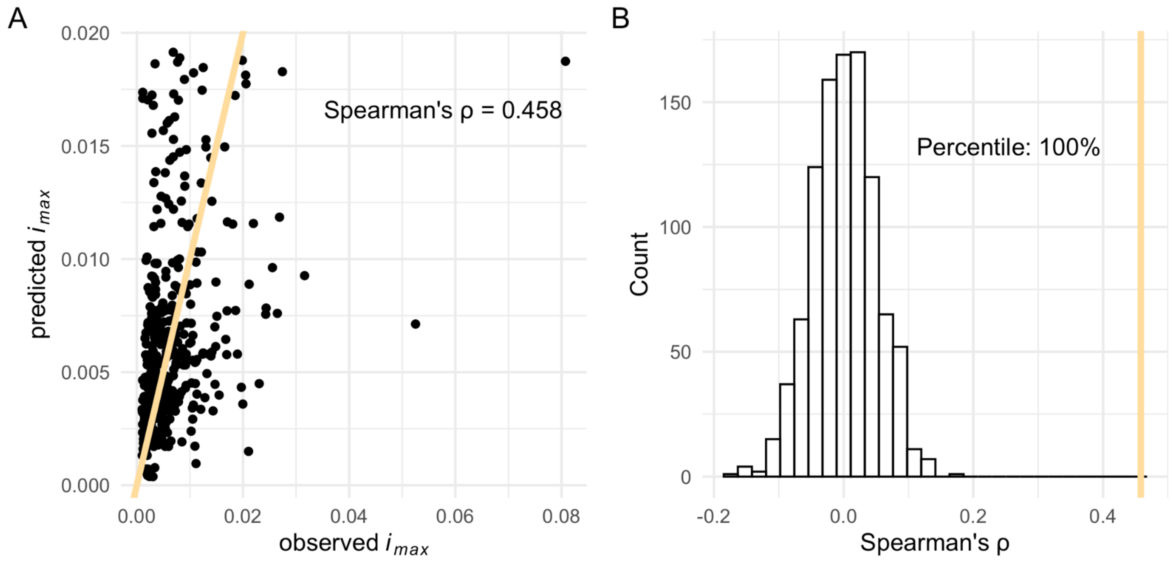

Figure S5. (A) Observed vs. predicted maximum incidence (i_{max}) for empirical epidemic outbreaks (N = 449). The yellow line represents the identity line (x = y). (B) Distribution of Spearman correlation coefficients between observed and predicted i_{max} from 1,000 permutations, where both the onset and municipality of the 449 epidemics were randomized. The actual observed correlation coefficient is shown as a vertical yellow line.

Supplementary tables

Table S1. Summary statistics describing the 250 parameter combinations with the lowest root mean squared errors from the parameter exploration with the Gaussian Process. These combinations represent the best-fitting sets of parameters for matching observed and predicted dengue maximum incidences across municipalities. IQR = Interquartile Range.

**Parameter**	**Min**	**Mean**	**Max**	**IQR**
Seasonality strength	6e-5	0.05	0.21	0.05
First case timing	0.00	0.50	1.00	0.32
Infectious period	4.00	5.02	6.00	0.97
Average mobility	1.00	2.51	5.00	1.67
Mobility skewness	0.05	0.48	0.95	0.40
Social structure	0.02	0.63	0.99	0.53
Family cluster size	1.16	11.17	19.90	8.71
Scaling factor	0.01	0.03	0.10	0.01
